# A novel transgenic mouse strain expressing PKCβII demonstrates expansion of B1 and marginal zone B cell populations

**DOI:** 10.1038/s41598-020-70191-y

**Published:** 2020-08-04

**Authors:** Ali A. Azar, Alison M. Michie, Anuradha Tarafdar, Natasha Malik, Geetha K. Menon, Kathleen J. Till, Nikolina Vlatković, Joseph R. Slupsky

**Affiliations:** 1 Department of Molecular and Clinical Cancer Medicine, University of Liverpool, 1st Floor Sherrington Building, Ashton Street, Liverpool, L69 3GE UK; 2 Molecular Lymphopoiesis Group, Institute of Cancer Sciences, College of Medical, Veterinary and Life Sciences, University of Glasgow, Glasgow, UK; 3 Department of Histopathology, Royal Liverpool University Hospital, Liverpool, UK; 4 Present Address: Barts Cancer Institute, Barts and the London School of Medicine and Dentistry, Queen Mary University of London, London, UK

**Keywords:** Chronic lymphocytic leukaemia, B cells, Chronic lymphocytic leukaemia

## Abstract

Protein kinase Cβ (PKCβ) expressed in mammalian cells as two splice variants, PKCβI and PKCβII, functions in the B cell receptor (BCR) signaling pathway and contributes to B cell development. We investigated the relative role of PKCβII in B cells by generating transgenic mice where expression of the transgene is directed to these cells using the Eµ promoter (Eµ-PKCβIItg). Our findings demonstrate that homozygous Eµ-PKCβIItg mice displayed a shift from IgD^+^IgM^dim^ toward IgD^dim^IgM^+^ B cell populations in spleen, peritoneum and peripheral blood. Closer examination of these tissues revealed respective expansion of marginal zone (MZ)-like B cells (IgD^+^IgM^+^CD43^neg^CD21^+^CD24^+^), increased populations of B-1 cells (B220^+^IgD^dim^IgM^+^CD43^+^CD24^+^CD5^+^), and higher numbers of immature B cells (IgD^dim^IgM^dim^CD21^neg^) at the expense of mature B cells (IgD^+^IgM^+^CD21^+^). Therefore, the overexpression of PKCβII, which is a phenotypic feature of chronic lymphocytic leukaemia cells, can skew B cell development in mice, most likely as a result of a regulatory influence on BCR signaling.

## Introduction

B cell receptor (BCR) signaling plays an essential role at critical stages of B cell development^1,2^. In the bone marrow spontaneous and environmental ligand-induced tonic signals from this receptor aid the transition of developing B cells from one stage of differentiation to the next until they emerge into peripheral circulation. In the periphery, mature B cells move between secondary lymphoid organs where specific antigen recognition by BCR results in their activation and differentiation to ultimately generate cells that yield specific antibody production and immune memory. Key signaling molecules have been found to be intricately linked to the stage specific responses of BCR signaling. While this is particularly true for the early stages of B cell differentiation where this relationship is well defined, the roles of signaling molecules involved in B cell fate decisions in the periphery are less well characterized and understood^2^.

One such signaling molecule is Protein kinase Cβ (PKCβ), which plays a central role in the appropriate regulation of B cell development and activation, including BCR signaling^3^. It has been demonstrated that knock-down of the PKCβ gene, *prkcb*, in mice results in immunodeficiency that is reminiscent of X-linked immunodeficiency, and corresponds to impaired humoral immune responses and B cell function^4^. PKCβ plays an important role in B cell development and activation, including BCR signaling^3,4^.

A key target of PKCβ during BCR engagement is Bruton’s tyrosine kinase (Btk). PKCβ phosphorylates Btk to provide feedback inhibition of BCR signaling by blocking its ability to activate phospholipase Cγ2 (PLCγ2) and stimulate Ca^2+^ release from intracellular stores^5^. Genetic studies have confirmed a functional relationship between PKCβ and Btk, showing that there is great similarity of B cell-related phenotypes resulting from targeted disruption of the genes coding for these proteins^4,6–8^. The gene coding for PKCβ, *PRKCB*, when transcribed is alternatively spliced to yield PKCβI and PKCβII, which differ from each other within their C-terminal 50 amino acids^9,10^. However, it is not clear whether these isoforms have redundant or specific functions in B cell development and activation.

Our previous studies of PKCβII in the malignant cells of B lymphoproliferative disorders have shown that overexpression of this PKC isoform is a phenotypic feature of chronic lymphocytic leukaemia (CLL) cells^11^. In addition, the Tcl1-transgenic mouse model of CLL demonstrates that PKCβ plays a key role in the pathogenesis of CLL, because PKCβ deficient mice do not develop CLL-like disease^12^. One possible explanation for this is the severe reduction in numbers of marginal zone B cells (MZ B cells) and B-1 B cells in PKCβ knock out mice^4^, since these are the cells from which CLL is thought to arise^13,14^. Interestingly, in virtually all mouse models of CLL, malignant cells develop from an expanded B-1 B cell compartment^15–19^, and this is similar to human disease which is preceded by a condition known as monoclonal B lymphocytosis^20^. Based on these combined findings, we wanted to investigate the effect of PKCβII overexpression in the B cell compartment. Considering the role of PKCβ in modulating BCR signaling and in B cell development, particularly the development of B-1 and MZ B cells, we hypothesized that transgenic expression of PKCβII within B cells of mice might lead to an expansion in populations of these B cell types.

## Materials and methods

### Generation of the Eµ-PKCβII tg mice

**Figure 1:**
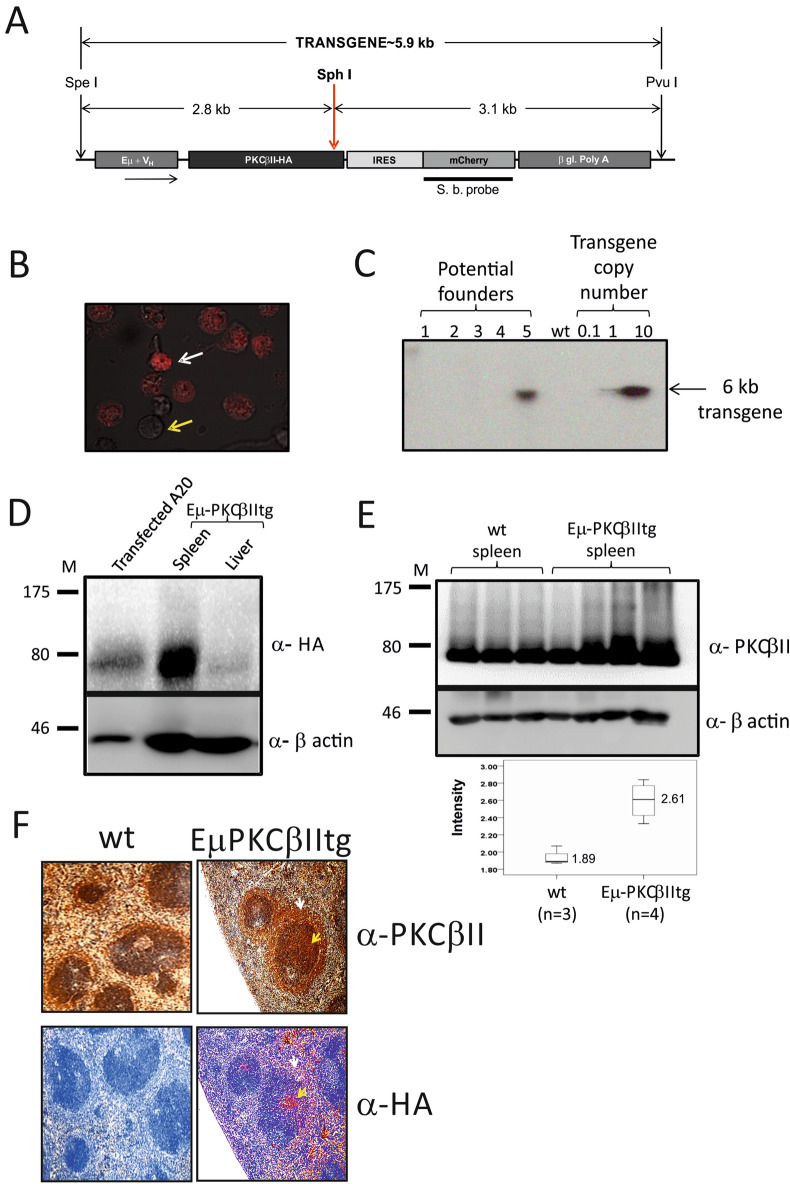
Generation of Eµ-PKCβII transgenic mice. (**A**) Schematic representation of the plasmid construct (pEµ-PKCβII-IRES-mCherry) used to generate transgenic mice. (**B**) A20 cell line transfected with pEµ-PKCβII-IRES-mCherry plasmid using nucleofection. White arrow indicates fluorescent A20 cells that were succesfully transfected and express mCherry. Yellow arrow indicates those cells which did not take up the plasmid. (**C**) Southern blot analysis of genomic DNA isolated from tail cuttings of transgenic progeny to identify potential Eµ-PKCβIIItg founder mice. Copy number of the inserted gene was estimated by the accompanying standard curve of plasmid DNA. (**D**) Immunoblot analysis of protein extracted from splenic and liver tissue of homozygous Eµ-PKCβIItg mice. Western blots were performed using antibodies against HA (*upper panel*) or β-actin (*lower panel*). As positive control, protein lysates prepared from A20 cells that had been transiently transfected with pEµ-PKCβII-IRES-mCherry. (**E**) *Upper and midde panel.* Respective western blot analysis of PKCβII and β-actin expression in splenic tissue of wt (n = 3) and Eµ-PKCβIItg (n = 4) mice. *Lower panel*. Comparison of PKCβII expression in wt and Eµ-PKCβIItg mice. Expression of PKCβII was quantitated relative to β-actin. (**F**) Immunohistochemical staining of spleen sections from Eµ-PKCβIItg and wt mice with anti-HA (stained with Fast Red) and anti-PKCβII antibodies (brown with DAB). With respect to Eµ-PKCβIItg mice, the spleen sections are sequential where upper and lower images can be superimposed using the white arrow which points to the MZ and the yellow arrow which points to the follicle. The images in parts (**C**–**E)** have been cropped to efficiently show the relevant details, uncropped images can be viewed in the Supplementary information accompanying this manuscript. All elements of this figure have been published in the PhD thesis of AAA^43^.

To generate Eµ-PKCβIItg mice, a pBSVE6BK plasmid (kindly provided by Dr. Raif Geha, Harvard Medical School) which exploits the VH promoter and the IgH-enhancer to specifically direct expression of target genes in B cells was used (pEµ vector^21^). The full length PKCβII coding sequence (2021-bp) was tagged with human influenza HA at its 3′ end by PCR, using the forward 5′-GAGAATCGATCAAGATG-GCTGACCCGGCTGCGGGGCC-3′, and the reverse 5′-GAGAGTCGACTCAAAGAGCGTAATCTGGAACATCGTATGGGTAGCTCTTGACTTCGGGTTTTAAA-3′ primers (Eurofins MWG Operon, London UK) prior to cloning into the ClaI (all restriction enzymes used in this study are from New England Biolabs, Hitchin UK) and SalI sites of the pEµ vector. An intra- ribosomal entry site (IRES) was sub-cloned into the BamHI and SalI sites of the multiple cloning site (MCS) of mCherry, and the IRES-mCherry coding sequence was then sub-cloned into pEµ-PKCβIIHA using Not-I and Apa-I placing it in a 5′→3′ unilateral orientation between the PKCβIIHA and β-poly-globin coding sequences in the Eµ vector (Fig. 1A). This construct was tested in the mouse B lymphoma cell line A20^22^ where 2 × 10^6^ cells were nucleofected™ with 2 µg pEµ-PKCβIIHA-IRES-mCherry using the solution V transfection kit and programme U-013 according to the manufacturer’s protocol (Lonza, Tewkesbury, UK) and fluorescence associated with the expression of mCherry observed (Fig. 1B). The construct containing pEµ-PKCβIIHA-IRES-mCherry was then digested with Pvu-I and Spe-I, DNA fragment purified and injected into pronuclei of zygotes isolated from C57Bl/CBA F1 hybrid mice. Mice generated from this procedure were screened for the presence of the transgene by Southern blot analysis on genomic DNA isolated from the tail and digested with SphI. Blots were hybridized with the mCherry specific DNA probe labeled with ^32^P-dCTP (Amersham Megaprime DNA Labelling System), essentially as described previously^23^. Founders were identified by higher expression, and/or evidence of a single integration site for the transgene (Fig. 1C), and those showing transgene integration in their genome were chosen for further breeding to generate the heterozygous and ultimately homozygous Eµ-PKCβIItg transgenic animals that were analysed in this study. To establish the first-generation of Eμ-PKCβII heterozygous mice, the founder mouse was backcrossed with C57BL/6 wild type mice to generate F1 progeny. The heterozygous mice from this crossing were then selected and inter-crossed to generate F2 progeny. Some of these F2 mice were homozygous, and, at this point, we reconsidered the line established and these mice were used for phenotypical characterization as well as for breeding to expand the line. Eµ-PKCβIItg, littermate wt and non-littermate control mice were all maintained under identical conditions. Mice were generated and bred in-house within the Biological Services Units at the University of Liverpool and the University of Glasgow. Animal experiments were performed with appropriate approval and licenses from the UK Home Office. All experimental procedures involving animals were performed in accordance to guidelines approved by Animal Welfare and Ethical Review Bodies at the University of Liverpool and University of Glasgow and were carried out in accordance with standard animal housing conditions under local and home office regulations.

### Western blot analysis

Proteins were extracted from tissue samples as described previously^23^. Briefly, snap frozen tissue samples were homogenised under liquid nitrogen using pestle and mortar. The homogenised tissue was resuspended in buffer containing 20 mM HEPES, 25% glycerol, 0.42 M NaCl, 1.5 mM MgCl_2_, 0.2 mM EDTA, 1 mM DTT, 1 mM PMSF and the following protease inhibitors: aprotinin (2 µg/ml), leupeptin (0.5 µg/ml), pepstatin A (1 µg/ml) and soybean trypsin inhibitor (100 µg/ml), subjected to 3 freeze/thaw cycles and then, following centrifugation, the supernatant was collected and protein concentration determined using a modified Bradford (BioRad, Hemel Hampstead UK). Equal amounts of protein were separated by SDS-PAGE, transferred on to Immobilon™ membranes (Millipore, Fisher Scientific UK Ltd, Loughborough, UK), and Ponceau-S staining was used to verify equivalent protein loading. The membranes were probed with the following antibodies according to established protocol^11^: PKCβII (C-18, Santa Cruz, Insight Biotechnology Ltd, Wembley UK) or HA [clone C29F4, (Cell Signaling Technologies, Hitchin, UK) or clone 16B12 (Covance, Cambridge Bioscience, Cambridge UK)] at a dilution of 1:1,000. Following incubation with secondary HRP-conjugated antibodies, protein bands were visualized using enhanced chemiluminescence (ECL) according to manufacturer’s protocol (GE Healthcare Life Sciences, Little Chalfont UK).

### Histopathology and immunohistochemistry

Splenic tissue was fixed in 10% neutral buffered formalin and then embedded in paraffin. To remove paraffin and rehydrate splenic sections a PT Link device (DAKO, Stockport UK) was used according to the manufacturer's instructions. Immunohistochemical staining was performed using a DAKO Auto-stainer automated slide processing system and the EnVision™ FLEX/HRP kit (DAKO, K8012). The primary antibodies used were PKCβII (C-18) and HA (C29F4). Hematoxylin and eosin (H&E) staining of splenic sections was carried out according to standard protocols.

### Cell preparation

Peritoneal cells were removed by injecting 5–10 ml ice cold modified PBS (1% BSA + 0.1% Sodium Azide, pH 7.2) into the peritoneal cavity followed by withdrawal of the peritoneal exudates. Spleens were removed and mechanically disrupted to prepare single cell suspensions. Erythrocytes in peripheral blood, peritoneal and splenic samples were lysed by treating with 0.165 M ammonium chloride, prior to washing the cell suspensions.

### Flow cytometry

Flow cytometry was employed to characterize mature B cell subsets in spleen, peritoneum and peripheral blood comparing Eµ-PKCβII tg mice with wt mice, as indicated. Cells (2–5 × 10^6^) were suspended in 200 µl modified PBS, then antibody cocktails were added and the cell suspension was incubated on ice for 30 min in the dark. Following this the cells were washed with 300 µl modified PBS, and then centrifuged at 2,000 rpm at 4 °C for 2 min. The supernatant was decanted and the pellet was re-suspended in 300 µl modified PBS. Labeled cells were acquired using a LSR-Fortessa flow cytometer (BD Biosciences) and analyzed using FACSDIVA™ and FlowJo™ (BD Biosciences, UK) software packages. Fluorescence-conjugated antibodies were purchased from BioLegend (London, UK) unless otherwise stated: Living Colors^®^ DsRed mCherry (PT3647-2, Clontech), CD45R/B220-PE (RA3-6B2), CD5-PE/Cy5 (53–7.3), IgD-APC (11-26c.2a), CD43-FITC (S11), IgM-APC/Cy7 (RMM-1), CD21/CD35-PE/Cy7 (7E9), CD24-PerCP/Cy5.5 (M1/69), FITC-Rat IgG2bκ Isotype Ctrl (RTK4530) and CD45R/B220-Alexa Fluor^®^ 488 (RA3-6B2). The percentage of B cell subsets, including FO (IgD^+^IgM^dim^), MZ (IgD^dim^IgM^+^CD43^neg^CD21^+^CD24^+^) and B-1 (B220^+^IgD^dim^IgM^+^CD43^+^CD24^+^) B cell populations were largely similar between wt littermate and non-littermate animals (data not shown), therefore non-littermate and littermate wt mice were considered a single group of wt mice.

### Calcium flux

Calcium flux analysis in isolated cells was carried out as described previously^24^. Briefly, splenic cells from Eµ-PKCβII tg hom/het mice were labelled with 1 μM Fura-2 AM (Invitrogen Ltd.) in buffer (145 mM NaCl, 5 mM KCl, 1 mM MgSO_4_, 1 mM CaCl_2_, 10 mM HEPES, 0·18% glucose and 0·2% BSA, adjusted to pH 7.4) at 37 °C for 30 min in the dark. Cells were washed, incubated with 10 μg/ml biotinylated anti-IgM for a further 30 min at 4 °C, washed and re-suspended at 1 × 10^6^/ml in buffer. Cells (1.5 × 10^6^) transferred to the fluorimeter cuvette were allowed to warm to 37 °C for 3 min prior to data acquisition. After recording basal fluorescence, 50 µl avidin was added to crosslink the BCR (final concentration 8.3 µg/ml) and recording continued for a further 3 min. Fluorescence was measured at 340 and 380 nm using a Hitachi F-7000 Fluorescence Spectrophotometer (Hitachi High Technologies America, Inc., Schaumburg, IL, USA).

### Ig concentration analysis

Blood was extracted from mice and serum was prepared by pelleting the RBCs at 12,000*g* for 10 min. The serum was aliquoted and stored at – 20 °C until needed. Assays of IgM concentration in serum were performed using the LEGENDplex™ kit (BioLegend, UK) following the manufacturers’ instructions. IgM concentrations were calculated using the LEGENDplex™ data analysis software dongle.

### Statistical analysis

All statistical analyses in this study were performed using GraphPad Prism^®^ 8 software.

## Results

### Characterization of Eµ-PKCβII transgenic mice

Based on the Southern blotting analysis, the number of pEµ-PKCβIIHA-IRES-mCherry transgene copies integrated in the single site of the founder mouse genome was estimated to be greater than one, but less than 10 copies (Fig. 1C). PKCβIIHA expression was then analysed by Western blot analysis and detected in spleen but not in liver of 6 month-old mice homozygous for the PKCβIIHA transgene (hereafter Eµ-PKCβIItg mice) (Fig. 1D), suggesting that transgene expression is tissue specific. A comparison of total PKCβII expression in protein extracts derived from the splenic tissue showed that PKCβII was expressed at significantly higher levels in Eµ-PKCβIItg mice compared with wt counterparts (Fig. 1E). In addition, analysis of HA expression within the spleen revealed that expression was concentrated within the follicle area of the peri-arteriolar lymphoid sheaths (PALS) and MZ, both of which are B cell rich areas (Fig. 1F). Although total PKCβII expression in the spleen of transgenic and wt mice showed a similar staining pattern, the intensity of staining was always greater in the tissue from transgenic mice where it correlated with that of HA. We were not able to detect the expression of mCherry in Eµ-PKCβIItg mice (data not shown). This may be because expression of a secondary gene from an IRES sequence can be variable and not always efficient in transgenic mice and therefore might have been below detection level^25^.

Eµ-PKCβIItg mice aged normally and did not show any signs of illness when aged up to 14 months. The WBC count of Eµ-PKCβIItg mice was in a normal range and did not differ from that in wt mice (Table 1). In addition, the spleen weight did not change significantly between Eµ-PKCβIItg mice and wt mice, and although there appeared a small but significant increased ratio of B cells to combined T/B lymphocytes in the spleen of EµPKCβIItg compared to wt mice, this ratio remained similar in the peripheral blood and peritoneum between these animals.

**Table 1:** Comparison of spleen weight, WBC count and B/B + T lymphocyte ratio in Eµ-PKCβIItg and wt control mice.

	Wild type	Std	Eµ-PKCβIItg	Std	P value
Spleen weight (mg)	129.4 n = 7	9.02	120.4 n = 9	5.9	0.63
WBC count (× 10^6^ per ml)	3.02 n = 7	0.78	3.74 n = 11	0.62	0.41
**B cell/B + T cells**					
Spleen	0.60 n = 10	0.046	0.73 n = 13	0.064	0.024
Peritoneum	0.85 n = 6	0.014	0.87 n = 10	0.024	0.25
Peripheral blood	0.71 n = 4	0.063	0.65 n = 10	0.052	0.44

B/B + T lymphocyte ratio in spleen, peritoneum and peripheral blood were determined using a FACS-based protocol identifying B cells (B220^+^CD5^−^ live lymphocytes) and T cells (B220^−^CD5^+^ live lymphocytes). Statistical analysis was performed using a Mann–Whitney U-test.

### Splenic tissue from Eµ-PKCβIItg mice display an expansion of the MZ B cell population and a concomitant reduction of the FO B cell population

**Figure 2:**
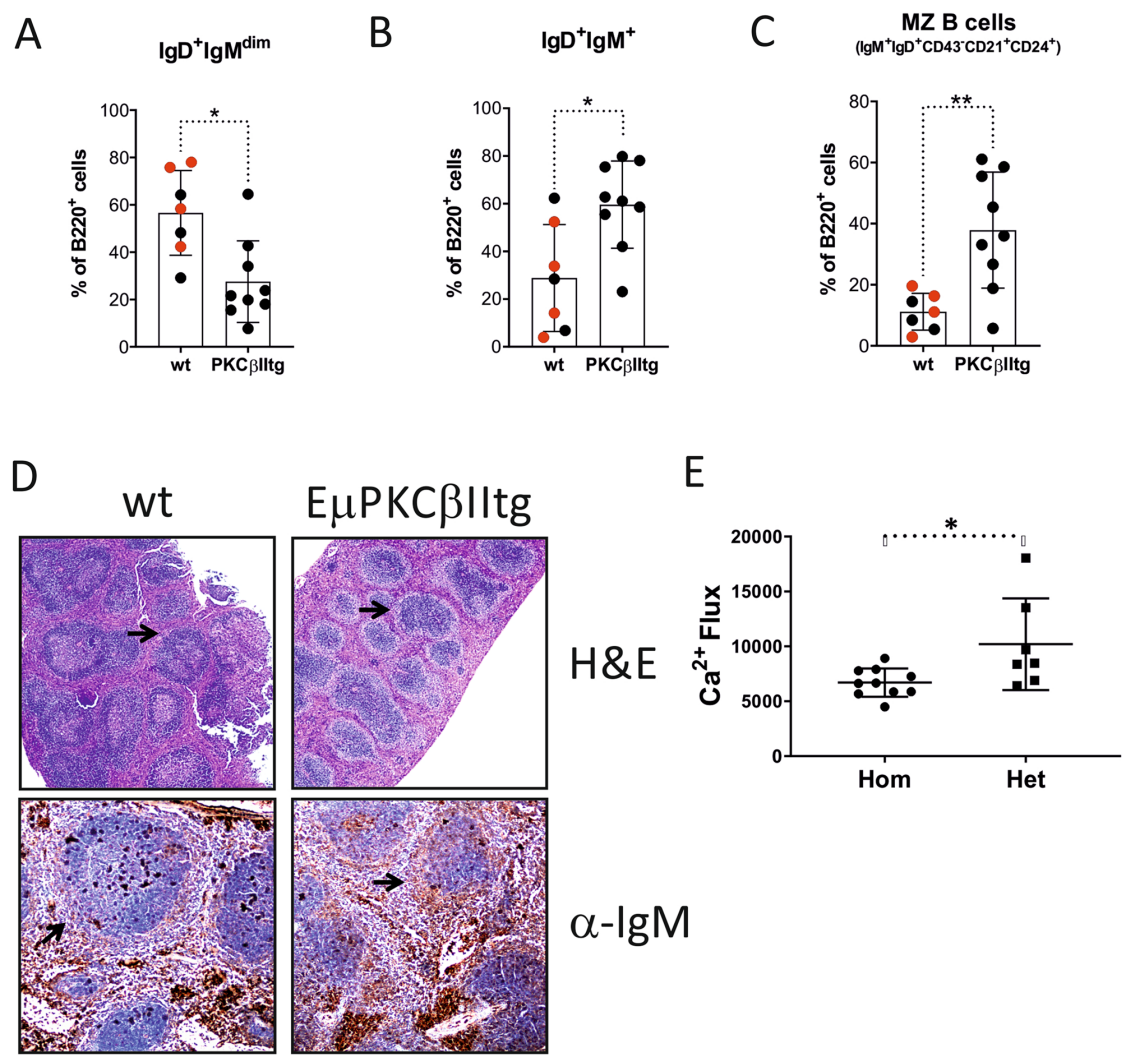
Effect of B cell-targeted expression of PKCβII on B cell populations in splenic tissue from Eµ-PKCβIItg and wt mice. Single cell suspensions prepared from spleens isolated from Eµ-PKCβIItg and wt mice were stained with a cocktail of antibodies containing B220, IgM, IgD, CD43, CD21 and CD24 markers, and then analysed by flow cytometry. Quantitative comparisons were then made of the percentage B220^+^ cells within the following gates (**A**) IgD^+^ IgM^dim^, (**B**) IgD^+^ IgM^+^, and (**C**) IgD^+^ IgM^+^ CD43^−^ CD21^+^ CD24^+^ (MZ-like B cell) for wt and Eµ-PKCβIItg mice as defined by the strategy illustrated in Supplementary Figure 2. Black dots in these graphs refer to wt and homozygous Eµ-PKCβIItg progeny derived from mating heterozygous Eµ-PKCβIItg mice. Red dots refer to wt mice with similar genetic backgrounds (C57BL/6) that are alike in terms of age to the transgenic mice. (**D**) *upper panels* H&E staining of splenic tissue from wt and Eµ-PKCβIItg mice. *Lower panels* anti-IgM staining of spleen sections from wt and Eµ-PKCβIItg mice. These images are representative of n = 2 experiments using splenic tissue from different mice that had been aged in excess of 12 months. Inset arrows indicate MZ. These histogram images have been published in the PhD thesis of AAA^43^. (**E**) BCR-induced Ca^2+^ flux in isolated splenic B cells from heterozygous and homozygous Eµ-PKCβIItg mice. Total flux was calculated as area under the curve is reported in arbitrary units. Statistical analysis for parts (**A**) (*P = 0.012), (**B**) (*P = 0.016), (**C**) (**P = 0.0052) and (**E**) (*P = 0.024) was performed using a Mann–Whitney U test.

Although total B cell counts were similar between EµPKCβIItg and wt mice, differences were observed between discrete populations. We applied the gating strategy shown in Supplementary Figure 2 for the analysis of B cells within splenic tissue from wt and Eµ-PKCβIItg mice. Thus, B220^+^ cells were first analysed for surface IgD and IgM expression. Eµ-PKCβIItg mice exhibited an increased percentage of IgD^+^ IgM^+^ B cells and decreased percentage of IgD^+^ IgM^dim^ B cells compared to wt control mice (Fig. 2A,B). Analysis of the IgD^+^ IgM^dim^ B cells identified them as mainly FO B cells because of their expression of CD21 and CD24, the phenotype of these cells seemed consistent between wt and Eµ-PKCβIItg mice (Supplementary Figure 2), despite lower numbers of these cells in the latter. The IgD^+^ IgM^+^ B cells were further gated for CD43 expression, and cells negative for this antigen were further analyzed for CD21 and CD24 expression. The major population identified, IgD^+^IgM^+^CD43^neg^CD21^+^CD24^+^, are “MZ-like” in phenotype and resemble a described precursor MZ cell population known as marginal zone precursor B cells^26,27^. This population of “MZ-like” B cells showed significantly greater representation in splenic tissue from Eµ-PKCβIItg compared to wt mice, suggesting a strong bias towards this phenotype in the former (Fig. 2C). The bias towards this MZ-like phenotype was further corroborated by H&E stains showing an extended MZ in Eµ-PKCβIItg compared with wt mice (Fig. 2D, *upper panels*), identified by anti-IgM staining (Fig. 2D, *lower panels*). Interestingly, this is the same area that seemed to stain strongly for PKCβII and HA in splenic tissue of transgenic mice (Fig. 1F). Of note, IgD^+^ IgM^+^ CD24^+^ CD43^neg^ cells negative for CD21 were present in splenic tissue from Eµ-PKCβIItg, but not from wt mice. This population may represent immature B cells but was not further analysed in this study. Functional analysis of splenic B cells from transgenic animals showed that BCR-induced Ca^2+^ flux was significantly suppressed in cells isolated from Eµ-PKCβIItg mice (Fig. 2E), an observation that is in line with the role of PKCβ in regulating the function of Btk and PLCγ2-induced calcium release^5^. Thus, these data suggest that B cell-targeted over-expression of PKCβII functionally restricts antigen receptor signaling resulting in an expansion of splenic MZ B cells in Eµ-PKCβIItg mice.

### The peritoneum of Eµ-PKCβII transgenic mice contains an elevated B-1 cell population

**Figure 3:**
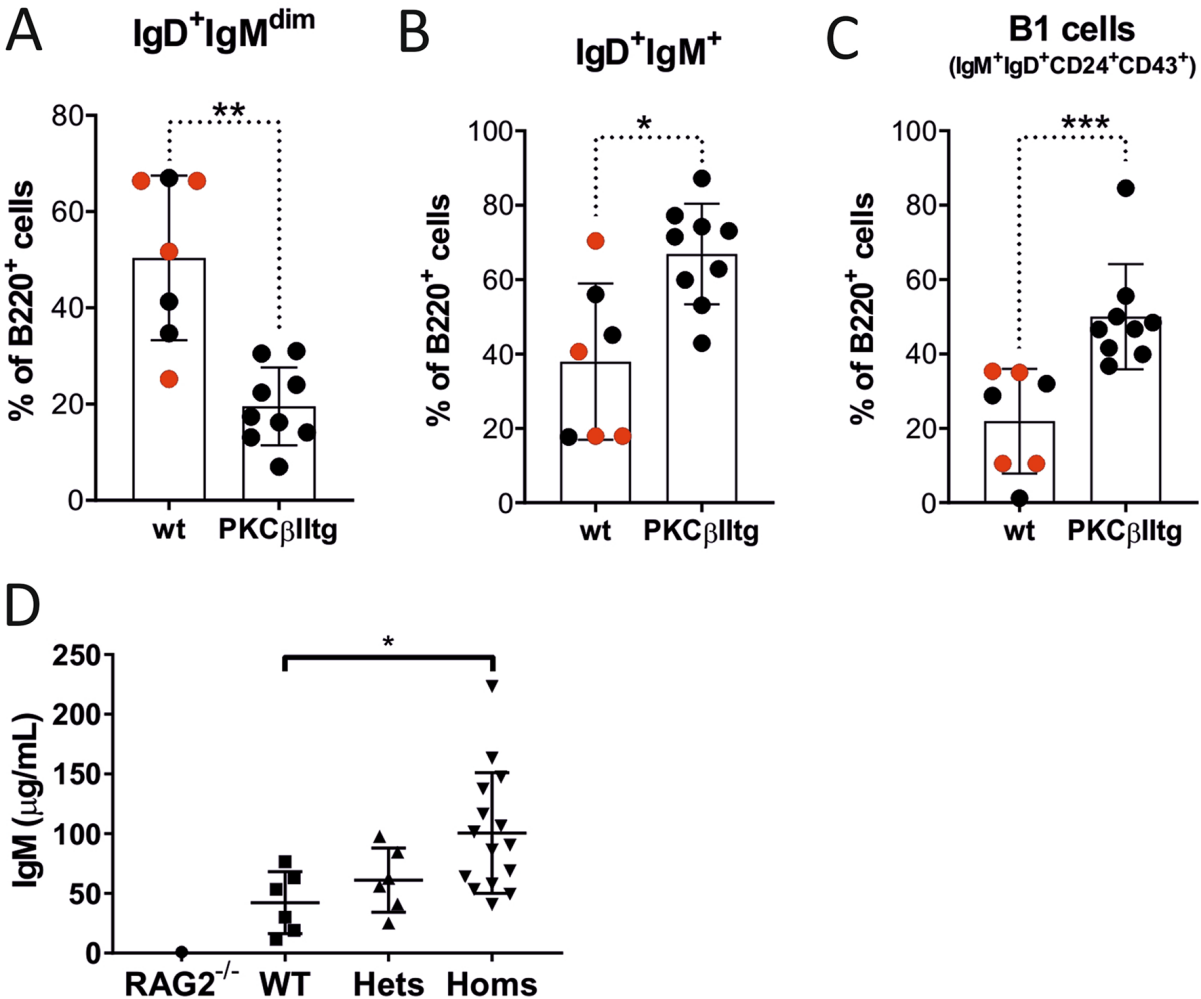
Effect of B cell-targeted expression of PKCβII on B cell populations in the peritoneum of Eµ-PKCβIItg and wt mice. Single cell suspensions prepared from peritoneal wash of Eµ-PKCβIItg and wt mice were stained with antibodies to B220, IgM, IgD, CD43, CD24, and CD5 and analyzed by flow cytometry. Quantitative comparisons were then made of the percentage B220^+^ cells within the following gates (**A**) IgD^+^ IgM^dim^, (**B**) IgD^dim^ IgM^+^, and (**C**) IgD^dim^ IgM^+^ CD43^+^ CD24^+^ (B-1 cells) for wt and Eµ-PKCβIItg mice as defined by the strategy illustrated in Supplementary Figure 3. Black dots in these graphs refer to wt and homozygous Eµ-PKCβIItg progeny derived from mating heterozygous Eµ-PKCβIItg mice. Red dots refer to wt mice with similar genetic backgrounds (C57BL/6) that are alike in terms of age to the transgenic mice. (**D**) Comparison of IgM levels in serum derived from Rag2 deficient, wt, and heterozygous and homozygous Eµ-PKCβIItg mice. Statistical analysis for parts (**A**) (**P = 0.0040), (**B**) (*P = 0.042), and (**C**) (***P = 0.0010) was performed using a Mann–Whitney U test, for part (**D**) analysis was done using a one-way ANOVA and Tukey’s multiple comparisons test (P = 0.040).

Peritoneal B220^+^ B cells exhibited a significant decrease in the percentage of IgD^+^ IgM^dim^ cells, coupled with significant increase in the percentage of IgD^dim^ IgM^+^ cells in Eµ-PKCβIItg mice when compared to wt mice (Fig. 3A,B, Supplementary Figure 3). Further analyses revealed that the populations of IgD^+^ IgM^dim^ cells defined by CD24 and CD43 expression were largely similar between wt and Eµ-PKCβIItg mice (Supplementary Figure 3). However, similar analysis of IgD^dim^ IgM^+^ cells showed that the proportion of CD24^+^CD43^+^ cells in Eµ-PKCβIItg mice was significantly increased compared to wt mice (Fig. 3C). These cells carry a B-1 B cell phenotype (B220^+^ IgM^+^ IgD^dim/−^ CD43^+^ CD24^hi^) and are likely to be B-1a cells because the majority of them are also positive for CD5 (Supplementary Figure 3). Taken together, these results suggest that B cell-targeted over expression of PKCβII results in accumulation of B-1a B cells in the peritoneum of Eµ-PKCβIItg mice.

### Serum IgM levels are increased in Eµ-PKCβIItg mice

Consistent with the increased proportion of MZ and B-1a cells observed in spleen and peritoneum, Eµ-PKCβIItg mice also exhibited elevated levels of serum IgM compared to wt animals (Fig. 3D). As a negative control we analysed serum derived from a single Rag1^−/−^ mouse to show the background of detection for the assay used.

### Peripheral blood from Eµ-PKCβIItg mice contains an expanded population of immature B cells

**Figure 4:**
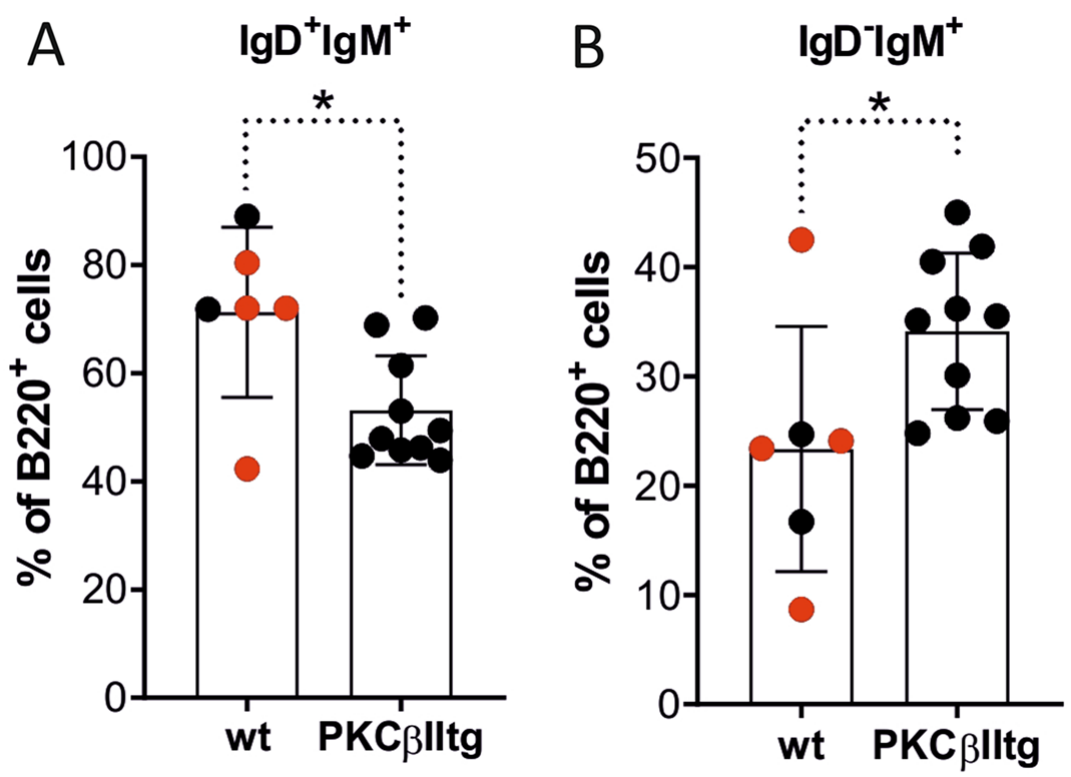
Effect of B cell-targeted expression of PKCβII on B cell populations in peripheral blood of Eµ-PKCβIItg and wt mice. Single cell suspensions prepared from peripheral blood and bone marrow of Eµ-PKCβIItg and wild type mice were stained with antibodies to B220, IgM, IgD, and CD21 and analyzed by flow cytometry. Quantitative comparisons were then made of the percentage B220^+^ cells within the following gates (**A**) IgD^+^ IgM^+/−^, and (**B**) IgD^dim^ IgM^+/−^ for wt and Eµ-PKCβIItg mice as defined by the strategy illustrated in Supplementary Figure 4. Black dots in these graphs refer to wt and homozygous Eµ-PKCβIItg progeny derived from mating heterozygous Eµ-PKCβIItg mice. Red dots refer to wt mice with similar genetic backgrounds (C57BL/6) that are alike in terms of age to the transgenic mice. Statistical analysis for parts (**A**) (*P = 0.030) and (**B**) (*P = 0.023) was performed using a Mann–Whitney U test.

When B220^+^ B cells derived from peripheral blood were analysed for IgD and IgM expression (Supplementary Figure 4), an expansion of IgD^dim^ IgM^+/−^ B cells was evident in Eµ-PKCβIItg mice compared to wt mice as was a reduction of IgD^+^ IgM^+/−^ B cells (Fig. 4). Analysis of CD21 expression on IgD^dim^ IgM^+/−^ B cells and IgD^+^ IgM^+/−^ B cells from Eµ-PKCβIItg and wt mice showed that the former were CD21^neg^ while the latter were CD21^+^ (Supplementary Figure 4). As CD21 is a marker of B cell maturity^28^, the lack of CD21 expression on IgD^dim^ IgM^+/−^ B cells from Eµ-PKCβIItg mice suggests that these cells are most likely immature B cells. Considering that the WBC and the B: B/T ratio in peripheral blood is not significantly different between Eµ-PKCβIItg and wt mice (Table 1), these results suggest that B cell-directed expression of PKCβII results in a shift towards expansion of the immature B cell population in peripheral blood.

## Discussion

Upregulated expression of PKCβII is frequently observed in the malignant cells of B lymphoproliferative diseases^29^, and, in particular, CLL where its overexpression is a phenotypic feature^11^. Because of this, we aimed to analyze the effect of PKCβII overexpression on the B cell compartment by generating a transgenic mouse strain where expression of PKCβII was driven by the Eµ promoter. Consistent with our initial hypothesis, the consequence of B cell-directed PKCβII overexpression is a proportional change in FO, MZ-like, B-1 and immature B cell populations respectively in the spleen, peritoneum and peripheral blood of Eµ-PKCβIItg mice. Further examination of the Eµ-PKCβIItg mice shows suppression of BCR signaling in splenic B cells, and elevated levels of serum IgM. Although expansion of the B-1 cell compartment is a feature of several mouse models of CLL^15–19^, we did not observe disease in Eµ-PKCβIItg mice and the proportion of B cells to combined B/T cells (taken as total lymphocytes) in these animals remained largely similar to that of wt mice despite aging them for 15 months. We conclude that PKCβII plays an important role in B cell development, with overexpression resulting in biased MZ-like and B-1 B cell development, but not in the pathogenesis of a CLL-like or other B lymphoproliferative disease in Eµ-PKCβIItg mice.

A limitation of this study is that our findings are based on data generated from analysis of animals that were derived from a single founder that was minimally backcrossed into the parental strain. The phenotype we describe could therefore potentially be a result of positional effect or an unidentified factor involved in producing the Eµ-PKCβIItg mice. However, comparison of B cell phenotypes within spleen, peritoneum and peripheral blood from the wt mice we analyzed are similar between littermate and non-littermate (i.e. C57BL/6 background) wt animals. These findings indicate that the genetic background of the strains used to generate Eµ-PKCβIItg mice had little, if any, effect on our observations. Furthermore, the phenomenon we observe is consistent with a mechanism whereby higher expression of PKCβII within B cells of Eµ-PKCβIItg mice could limit BCR signaling by suppressing Btk activation. A well-described role of PKCβ in B cells is the phosphorylation of the S^180^ residue in Btk leading to downregulation of its function^4,5,30^. Our own work shows that overexpressed PKCβII in primary CLL cells limits BCR signaling in the presence of pS^180^^11,31^. The novel mouse model we describe herein recapitulates these findings and we show that BCR signaling in B cells from Eµ-PKCβIItg mice is suppressed. In contrast, a study describing the phenotype of PKCβ knockout mice showed enhanced BCR-mediated signaling in B cells from these animals^4^, a phenomenon similar to that observed in a study of PKCβ inhibition on BCR signaling^32^. This same study also showed that MZ and B1 B cell populations, as well as serum IgM levels were severely reduced in PKCβ knockout mice^4^. Together, these observations provide support to our interpretation that suppressed BCR signaling resulting from targeted PKCβII overexpresson in Eµ-PKCβIItg mice leads to expansion of MZ-like and B1 B cells within total B cell populations and increased serum IgM levels. Further complementary support can be found in our observation that splenic B cell populations are slightly increased in Eµ-PKCβIItg mice, whilst Leitges et al.^4^ observed a slight decrease in these populations in their PKCβ knockout mice. Taken together, these observations suggest that the phenotype we observe is most likely due to transgenic overexpression of PKCβII rather than a confounding factor.

In the current study we have focused on PKCβII and have not considered its splice variant PKCβI. Since we have not silenced the expression of the gene that codes for PKCβ in our system, we cannot rule out the contribution of PKCβI to the phenotype we have generated. Future studies crossing the Eµ-PKCβIItg mouse with a PKCβ null mouse would enable us to determine the role of PKCβII in B cells more precisely.

It is intriguing that despite the noted lineage biases observed in B cell populations in the Eµ-PKCβIItg mice, aged animals did not develop a disease endpoint. A possible reason for this could be that overexpression of PKCβII alone is not sufficient to drive the pathogenesis in B lymphoid diseases. However, Eµ-PKCβIItg mice may be more susceptible to the development of these diseases since it has been demonstrated that, for example, in colon cancer, overexpression of PKCβII promotes development of disease by increasing the sensitivity of colonic epithelial cells to carcinogenic stimuli^33,34^. Indeed, this may be particularly relevant for CLL because this disease appears to develop from a lymphocytosis of B-1 cells in both mice and humans^14–17^. If this hypothesis is correct, Eµ-PKCβIItg mice crossed with another mouse model of CLL, such as the Tcl-1 model, would exhibit accelerated development of disease because of the expanded population of B-1 cells. An alternative reason for the lack of disease development may be that the expression levels of PKCβII achieved in our mouse model are not high enough to facilitate neoplastic transformation. Our previous work in CLL showed that the malignant cells in this disease express up to sevenfold greater amounts of this PKC isozyme than do normal B cells^17^. In the current study we were only able to achieve an approximate twofold increase of PKCβII in Eµ-PKCβIItg compared to wt mice.

We propose that the modest increase in protein levels of PKCβII observed in Eµ-PKCβIItg mice alters BCR signaling resulting in skewed representation of B cell population subsets in the periphery with expansion of MZ-like cells, B-1 B cells and immature B cells as we describe in this study. PKCβII in B cells has two key targets; CARMA1 (CARD11) which, when phosphorylated, combines with MALT1 and Bcl10 to form a complex that recruits TAK1 to stimulate activation of the NFκB and JNK signaling pathways and promote cell survival^35–37^, and Btk where S^180^ phosphorylation downregulates its function as discussed above^5^. Hence, overexpression of PKCβII will preserve survival of B cells while limiting their ability to respond to BCR engagement. Such limitation is likely to affect B cell differentiation and our observations can be explained by the "strength of signal" model suggested by Cariappa et al.^38,39^ where transitional B cells in the periphery respond to strong or "triggered" signals delivered via the BCR with commitment to FO B cell differentiation, while weaker or "tickled" BCR signals lead to differentiation towards MZ and B-1 B cells. The absence of BCR signals results in cell death due to neglect. This is likely the explanation for the phenotype of the MZ-like B cells we identify. These cells possess similarity to MZ precursor B cells^26^, the presence of which are directly related to BCR signaling strength as is demonstrated in Aiolos- and CD22-deficient mice^27,38,40^. Weakening of BCR signaling by overexpression of PKCβII is also likely to be responsible for the increased number of immature B cells in peripheral blood we observe in Eµ-PKCβIItg mice. Elimination of self-reactive B cell clones occurs at the pre-B cell stage within bone marrow, and is achieved either by receptor editing whereby B cell development is arrested while further gene rearrangements occur in order to mitigate self-reactivity^41^, or by the induction of apoptosis by strong BCR signals^42^. Since both of these processes are driven by Btk^8^, overexpression of PKCβII within pre-B cells of Eµ-PKCβIItg mice could limit strong self-antigen-driven BCR signaling by suppressing Btk activation thus generating the increased numbers of immature B cells seen here. We propose that the modestly increased levels of PKCβII in Eµ-PKCβIItg mice alters BCR signaling such that B cell subsets become skewed in the periphery as demonstrated; populations of MZ-like cells, B-1 B cells and immature B cells all expand^5,8,26,27,35–42^.

In conclusion, we have generated a transgenic mouse strain with targeted overexpression of PKCβII within B lineage cells; Eµ-PKCβIItg. We show that the phenotype associated with Eµ-PKCβIItg mice skews development of MZ-like and immature B cells over FO B cells in the spleen and peripheral blood, and of B-1 B cells in the peritoneum, thereby providing in vivo data that support our hypothesis that overexpression of PKCβII might result in an expansion of B1 and MZ B cell populations. Considering the phenotype of the PKCβ knockout mouse^3^, we have speculated that the increased populations of MZ-like and B-1a cells within the Eµ-PKCβIItg mouse would generate a phenotype where IgM antibodies would be prevalent and this was indeed the case. Thus, we have demonstrated that PKCβII specifically plays an important role in B cell development in mice. This novel mouse strain may be useful in studies of diseases involving BCR signaling, and, in particular, CLL.

## Supplementary information

Supplementary Information 1.
