# Supplementary material for: A novel transgenic mouse strain expressing PKCβII demonstrates expansion of B1 and marginal zone B cell populations
Source: Sci Rep. 2020 Aug 4;10:13156. doi: 10.1038/s41598-020-70191-y (PMC7403146; doi:10.1038/s41598-020-70191-y)

Supplementary Information

# A novel transgenic mouse strain expressing PKCβII demonstrates expansion of B1 and marginal zone B cell populations.

Ali A. Azar, Alison M. Michie, Anuradha Tarafdar, Natasha Malik, Geetha K. Menon, Kathleen J. Till, Nikolina Vlatkovic and Joseph R. Slupsky

Supplementary Information

**Figure 1C**: Uncropped full length Southern blot image. The inset box illustrates how the image was cropped to produce Figure 1C within the manuscript.

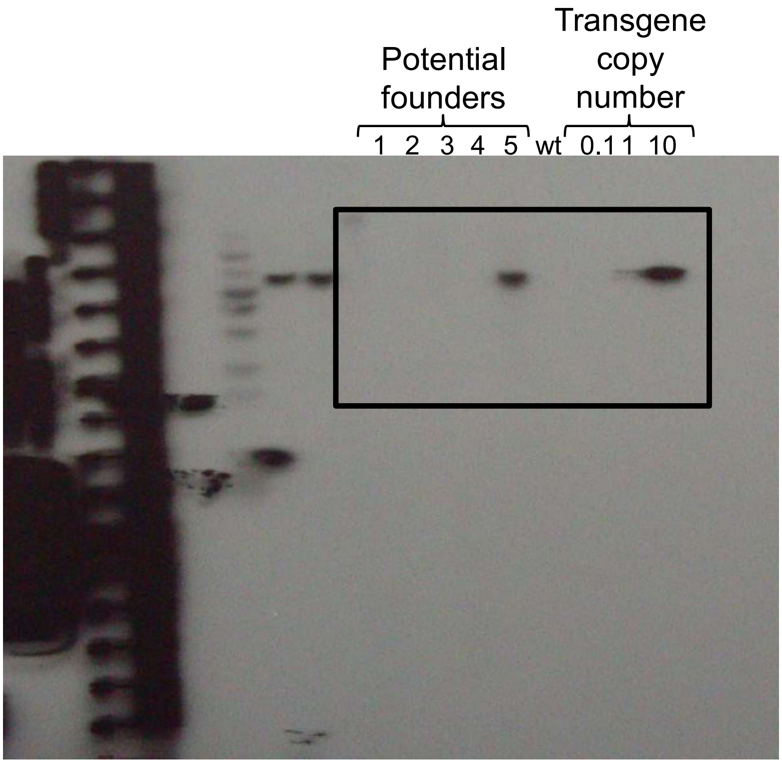

Supplementary Information

**Figure 1D**: Uncropped full length western blot (probed with α-HA antibody) of top image. The inset box illustrates the lanes taken to produce Figure 1D within the manuscript.

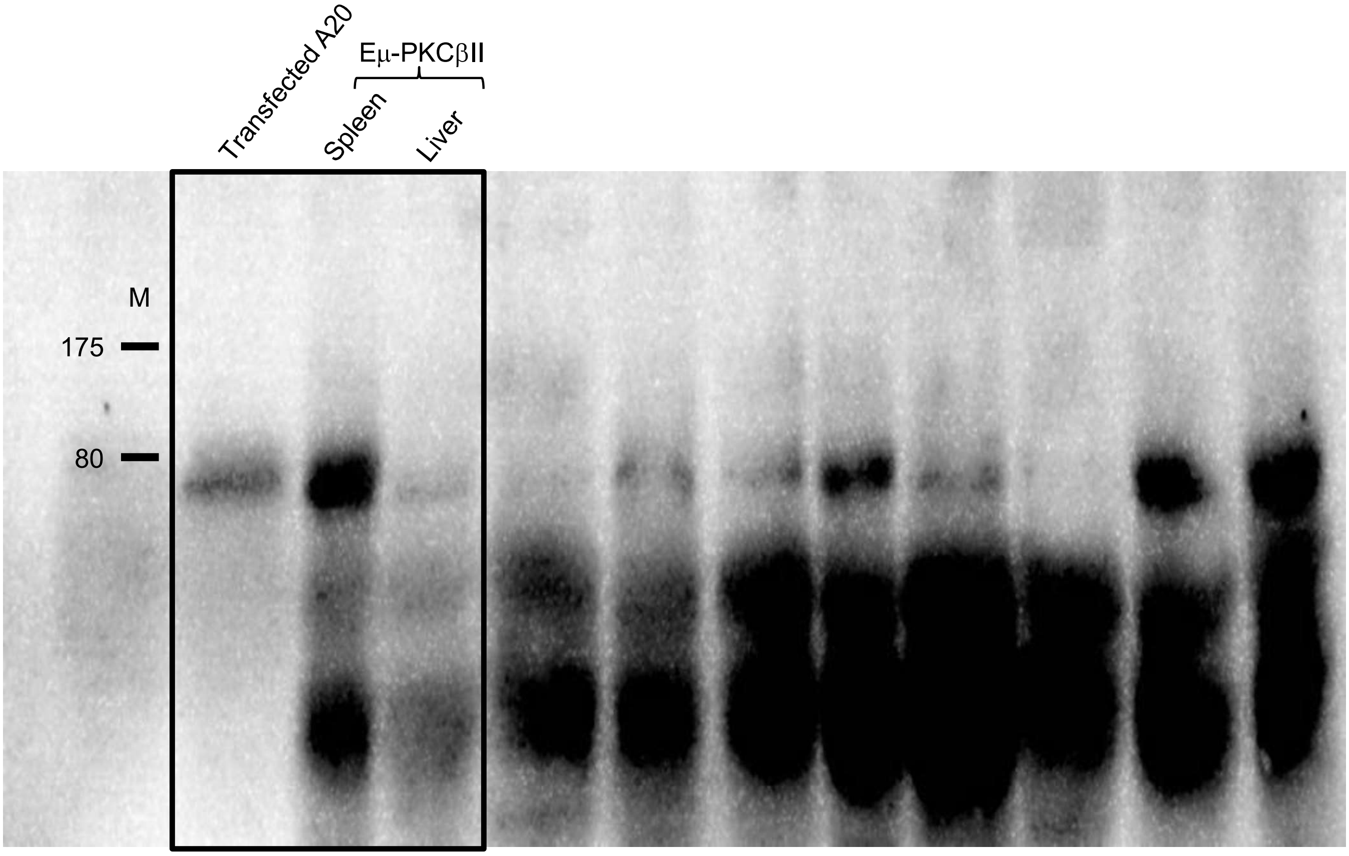

## Supplementary Information

**Figure 1D**: Uncropped full length western blot (probed with α- β actin antibody) of bottom image. The inset box illustrates the lanes taken to produce Figure 1D within the manuscript.

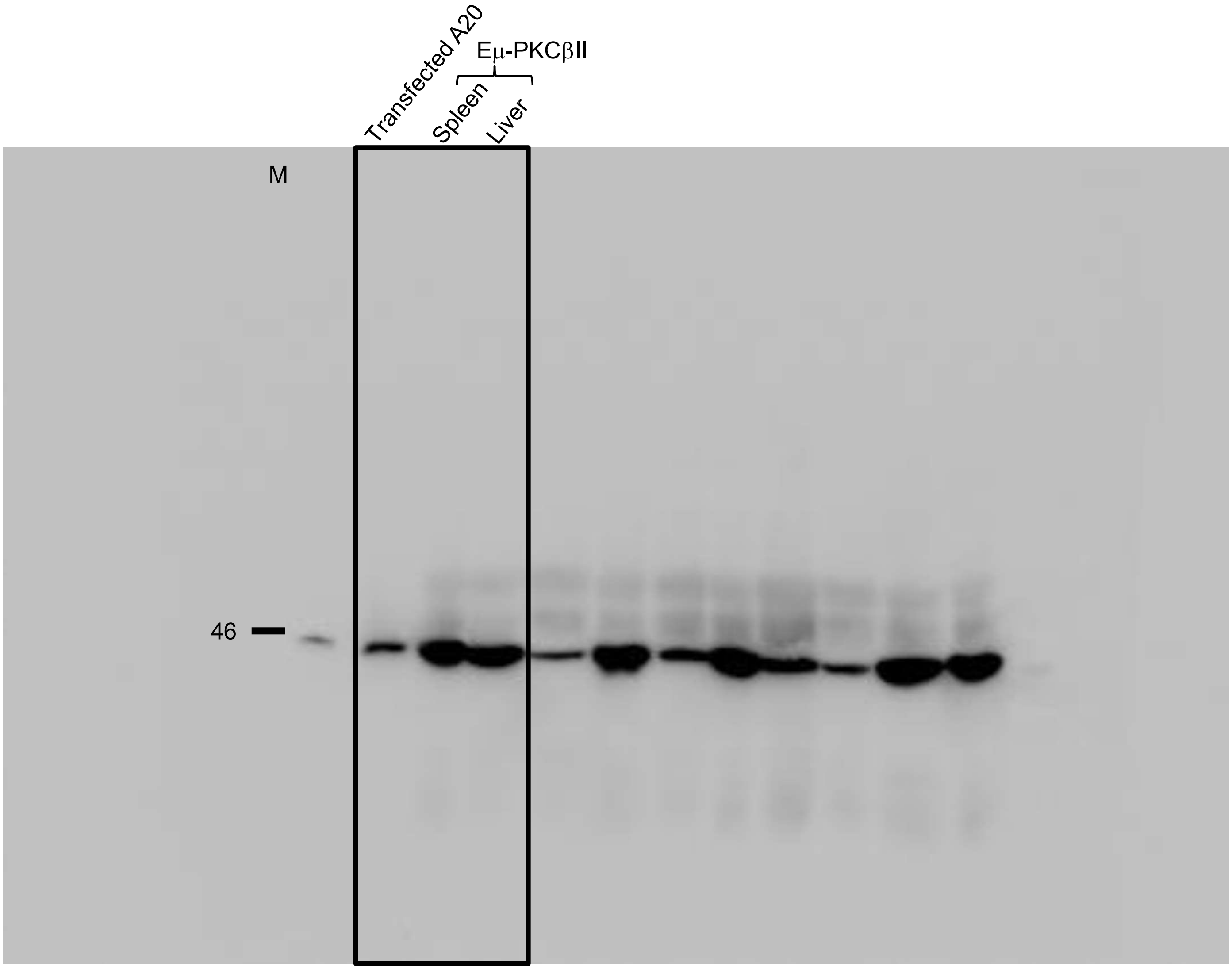

Supplementary Information

**Figure 1E**: Uncropped western blot (probed with α-PKCβII antibody) of top image. The inset box illustrates the lanes taken to produce Figure 1E within the manuscript.

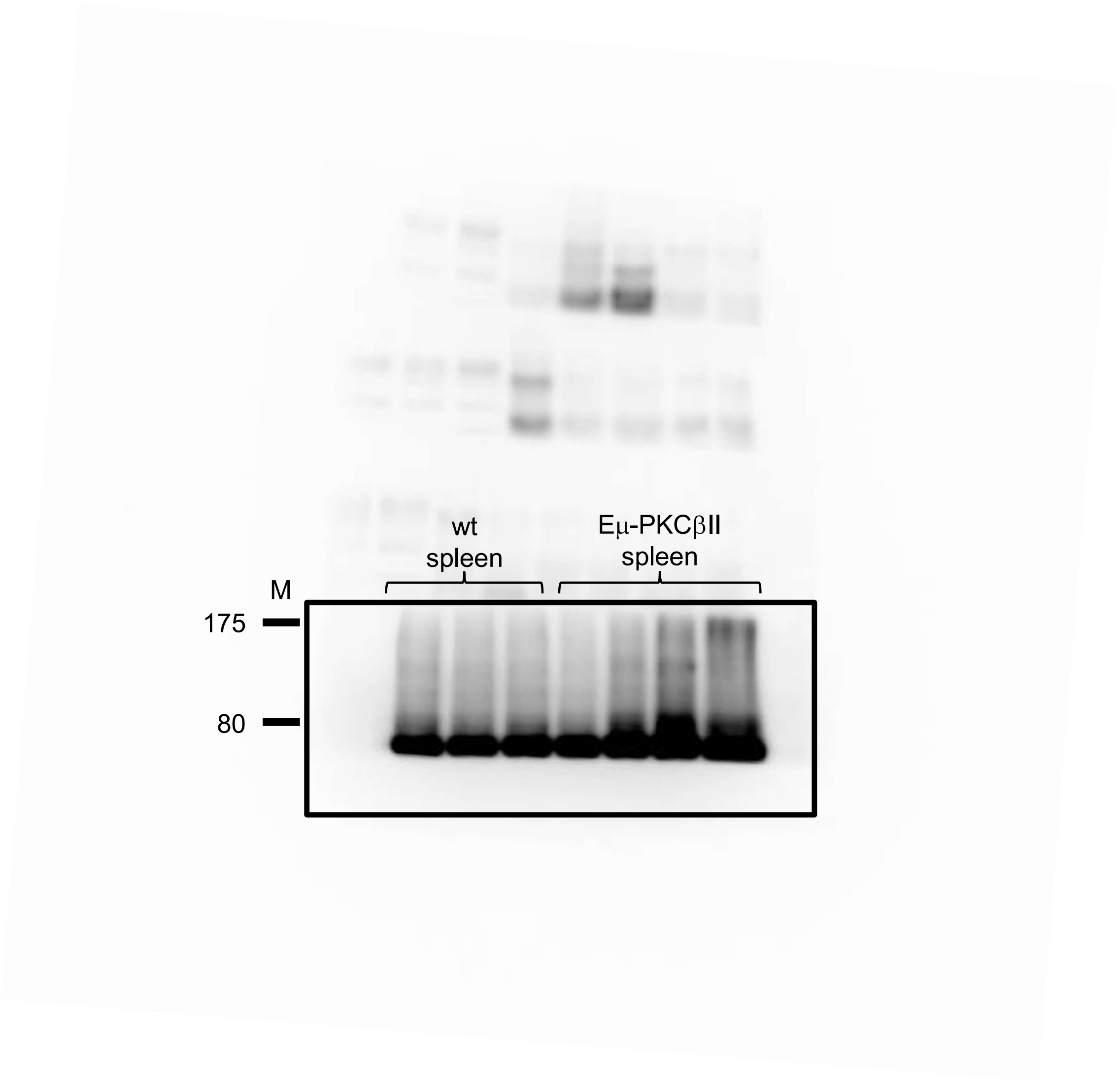

# Supplementary Information

**Figure 1E**: Uncropped western blot (probed with α- β actin antibody) of bottom image. The inset box illustrates the lanes taken to produce Figure 1E within the manuscript.

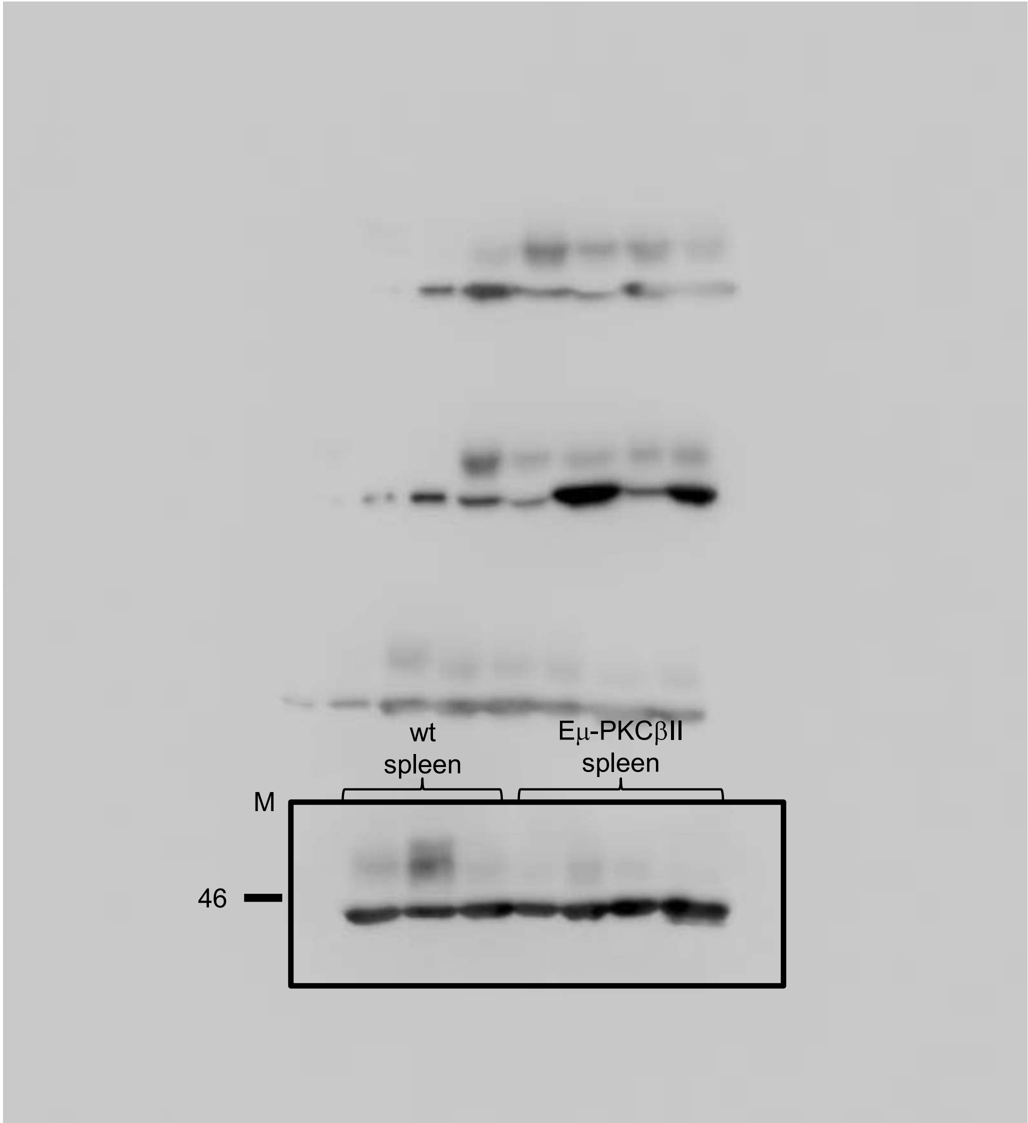

# Supplementary Information

**Figure 2**: *<u>Gating strategy employed to discriminate MZ and FO B cells within splenic tissue from wt and Eμ-PKCβIItg mice</u>*. Single cell suspensions from mechanically disrupted spleens isolated from wt (upper dot plots and histograms) and Eμ-PKCβIItg (lower dot plots and histograms) mice were first gated by FSC and SSC to identify single cells. Lymphocytes were identified within single cells by B220 strong positivity. Cells were then gated based on surface IgD and IgM expression to identify B cells, and then on CD43. $IgD^{dim}/IgM^{+}/CD43^{neg}$ cells were identified as MZ B cells based on strong CD24/CD21 expression. FO B cells were identified within $IgD^{+}/IgM^{-}/CD43^{neg}$ populations and demonstrated weaker expression of CD24/CD21 than did MZ B cells.

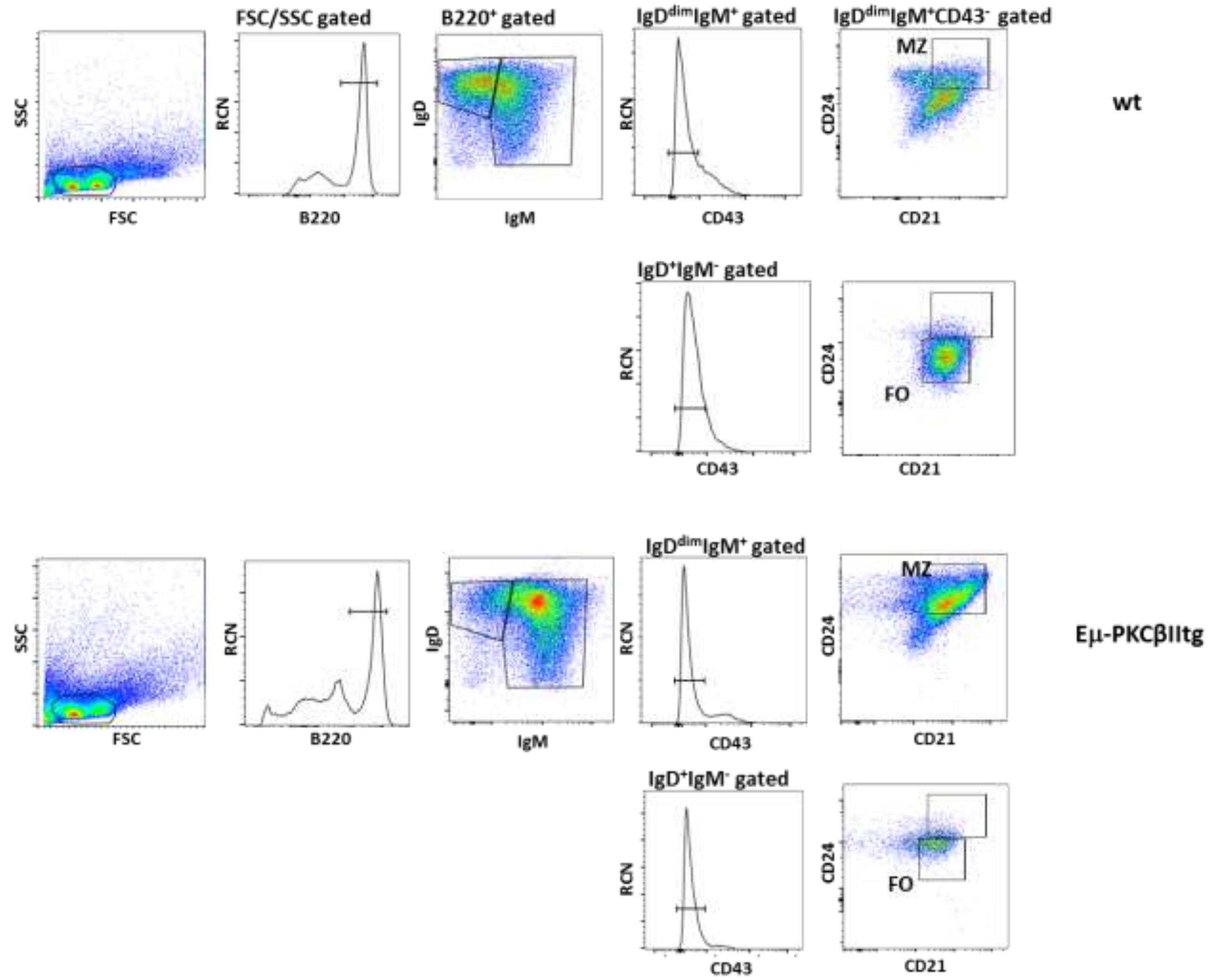

# Supplementary Information

**Figure 3**: <u>*Gating strategy employed to discriminate B1 B cells within the peritoneum from wt and Eμ-PKCβIItg mice*</u>. Single cell suspensions from peritoneal exudates isolated from wt (upper dot plots and histograms) and Eμ-PKCβIItg (lower dot plots and histograms) mice were first gated by FSC and SSC to identify single cells. Lymphocytes within these suspensions were identified by B220 strong positivity. Cells were then gated based on surface IgD and IgM expression to identify B cells, and then on CD43 to identify B1 B cells which were IgD$^{dim}$/IgM$^{+}$/CD43$^{+}$. B1 B cell identification was additionally confirmed by IgM and CD5 expression on B220$^{+}$ cells. IgD$^{+}$/IgM$^{dim}$ cells were also investigated for CD24 and CD43 expression.

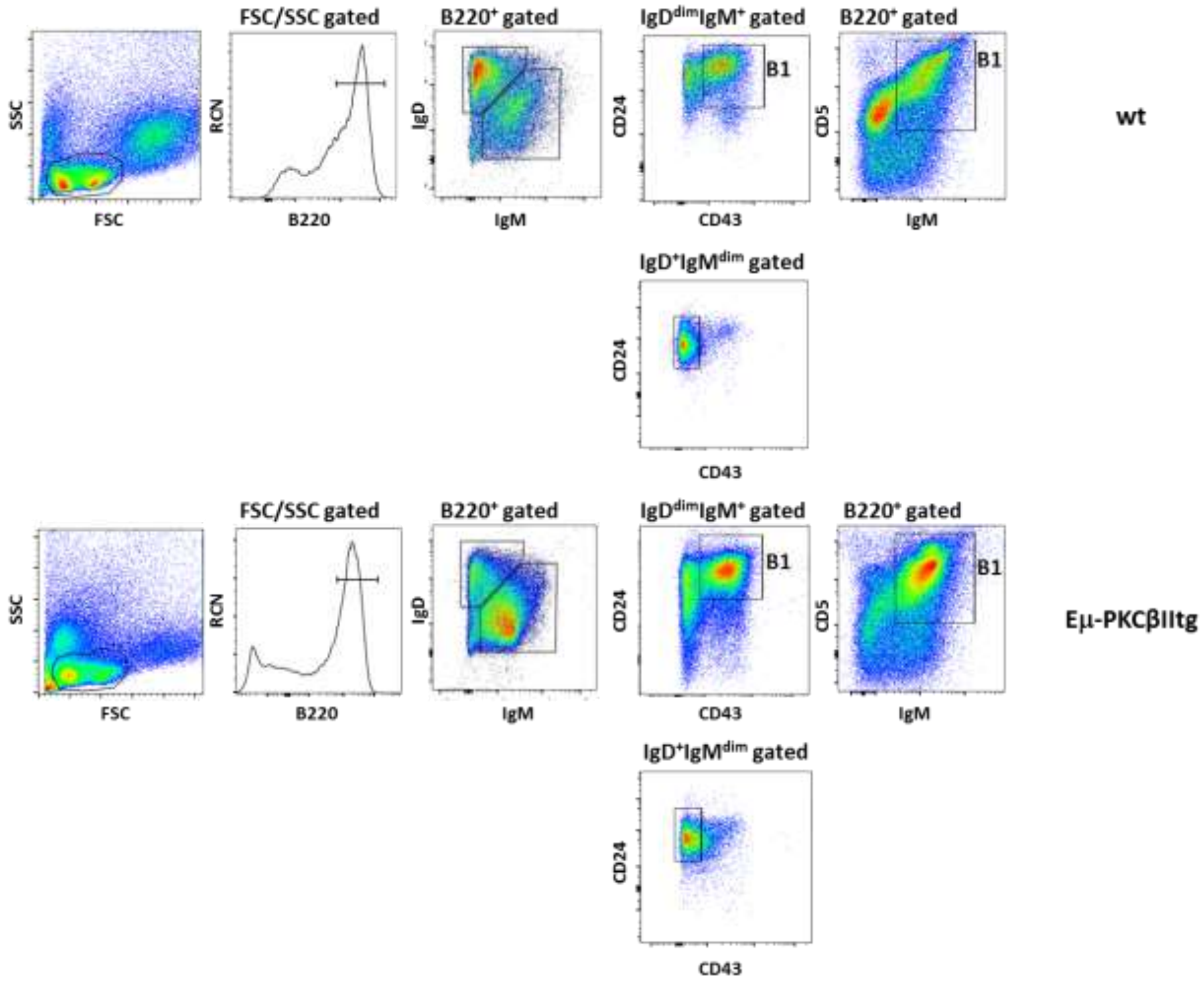

# Supplementary Information

**Figure 4**: <u>*Gating strategy employed to discriminate immature B cells within peripheral blood from wt and Eμ-PKCβIItg mice*</u>. PBMCs isolated from wt (upper dot plots) and Eμ-PKCβIItg (lower dot plots) mice were first gated by FSC and SSC to identify single cells. Lymphocytes within these suspensions were identified by B220 strong positivity. Cells were then gated based on surface IgD and IgM expression to identify B cells. Mature (*pink histograms*) and immature B cells (*blue histograms*) were identified by CD21 expression within IgD$^{+}$/IgM$^{+/-}$ and IgD$^{dim/-}$/IgM$^{+/-}$ populations, respectively.

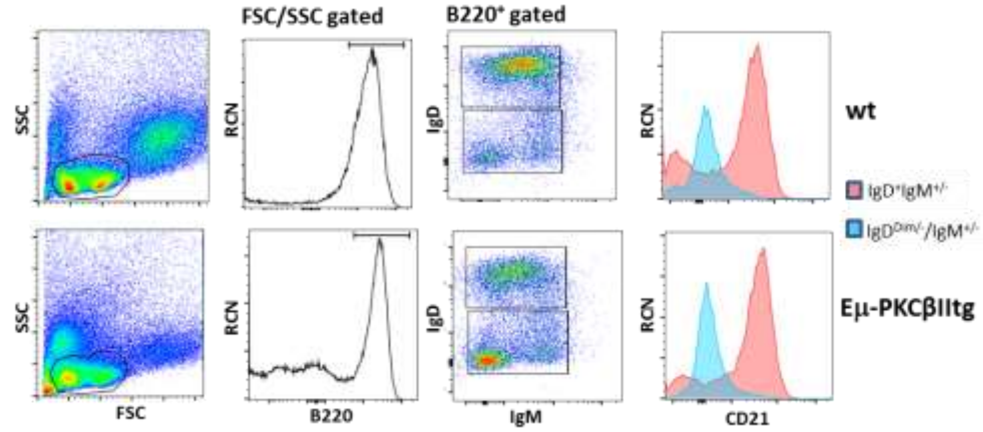

Supplement: Supplementary file 1 — Supplementary Information 1. [file 41598_2020_70191_MOESM1_ESM.pdf]
